# Monitoring Edentulism in Older New Zealand Adults over Two Decades: A Review and Commentary

**DOI:** 10.1155/2012/375407

**Published:** 2012-08-09

**Authors:** William Murray Thomson

**Affiliations:** Sir John Walsh Research Institute, Faculty of Dentistry, The University of Otago, Dunedin 9054, New Zealand

## Abstract

Historically, New Zealand has had the highest rates of edentulism in the world, but that rate has been falling quickly in recent decades. In 1997, projections were made for edentulism prevalence among 65–74-year-olds using national survey data from 1976 (where it was 72.3%) to 1988 (58.6%). That process assumed a logistic decline in edentulism, given that it would never have been 100% and will never get to 0%. This paper examines the validity of the projections using the estimate (29.6%) from the third national oral health survey, conducted in 2009 and considers the implications of this fall.

Edentulism is the state of having lost all of one's natural teeth [1]. It is conceptually distinct from the more common incremental loss of teeth which tends to occur throughout adult life [2], in that the transition to edentulism involves an explicit decision to undergo complete removal of the dentition (or what remains of it) in a single operation. That process usually involves the removal of some intact functioning teeth, meaning that the decision to opt for a full clearance is likely to be as much a social decision as it is a clinical one. Thus, the reasons for edentulism are complex, being both disease-related and societal [3], and this is reflected in well-documented international variations in the state's occurrence. For example, a major finding of the first international collaborative study was that the prevalence of edentulism did not appear to be associated with rates for dental caries and periodontitis in the participating countries [4], at least at the cross-national level. As Sussex highlighted in his comprehensive 2008 review [1], this underlined the strong influence of social (lay) and professional norms in edentulism occurrence. Only Scotland and Australia had anywhere near the same prevalence of edentulism, and countries outside the British Commonwealth had, on average, considerably lower rates. The former experienced marked declines in edentulism during the latter half of the 20th Century, but New Zealand had the highest initial rates and appeared to be the slowest to show the decline: the 50% edentulism prevalence rate among adults aged 21+ in 1950 had declined to only 47% by 1968. A degree of caution should be exercised in interpreting those estimates, however, because they did not come from national surveys. The general finding was later confirmed in the first New Zealand national oral health survey, conducted in 1976 [5], and that study underlined a strong association with socioeconomic status (SES), with rates among low-SES adults being considerably higher than among those of higher SES.

Epidemiological investigations of the occurrence of edentulism have shown variations by a number of characteristics and behaviours [1]. Sociodemographic differences are apparent, with rates being higher among females, older people, and those of lower SES or who belong to ethnic minority groups. Particular behaviours are also associated with edentulism prevalence, with higher rates among smokers and those with a problem-oriented dental visiting pattern. The recent review by Sussex [1] remains the most comprehensive and detailed one yet published, and it emphasised the importance of both social and disease-related influences on the occurrence of edentulism. The actors in the process are the patient and the dentist; the former is influenced by lay culture and values (both societal and personal), whereas the latter is influenced by his/her professional culture and values (notwithstanding the values of the particular society in which he/she is operating). An early analysis of US data found that patients' and dentists' values and beliefs were more important than clinical status in determining whether teeth were extracted [6]. That particular work concentrated on incremental (rather than total) tooth loss, but the principle is germane to considerations of the transition to edentulism. In relation to professional norms and values, the extraction-denture culture evident in New Zealand in the early to middle 20th century has been attributed partly to the relatively late maturing of dentistry as a *bona fide* profession in New Zealand [1], and that the profession's less-developed sense of autonomy at that time made its members more susceptible to pressure from patients and their families to perform full clearances [5].

For many decades, rates of edentulism among New Zealand (New Zealand) adults were among the highest in the world [1]. Countries such as Australia [3] also had relatively high edentulism rates during that period, but historical data indicate that New Zealand had the highest rates [4, 7]. Edentulous people have been shown to have poorer diets and associated nutrition than those with natural teeth [8, 9]. For example, an analysis of US NHANES data demonstrated that daily intakes of carrots, salads, and dietary fibre were lower among edentulous persons than those who were fully dentate. These dietary differences were reflected in their nutrition, with lower serum beta carotene, folate, and vitamin C levels than among their dentate counterparts. Not only are there nutritional disadvantages to being edentulous, the day-to-day lives of edentulous people are affected by having no teeth. As long ago as 1994, Slade and Spencer demonstrated poorer oral-health-related quality of life among edentulous older people, especially in relation to the domains of chewing and eating [10]. They were more likely to avoid eating some foods, to have sore spots and painful gums, and to find their food less flavourful. Thus, edentulism has both nutritional and social consequences; it is not a particularly benign state.

Monitoring the occurrence of an oral “end state” such as edentulism is important because it is an incontrovertible indicator of the functioning and adequacy of a country's oral health care system. Other factors being equal, a country with a considerably higher edentulism rate than another would be viewed as having a less equitable or appropriate oral health care system. National oral health surveys are the gold standard for such monitoring because they produce generalisable estimates of the conditions under investigation, and this (in turn) allows monitoring of trends in those conditions [11]. National surveys are complicated and expensive to conduct, and convincing health policy-makers to fund them can be a difficult task. New Zealand has now had three national oral health surveys. These were conducted in 1976 [12], 1988 [13], and 2009 [14]. The aim of this review paper is to describe and comment upon the changes in edentulism observed among older adults in those three surveys, and to examine the validity of predictions made [15] more than a decade before the third national oral health survey. Making predictions—let alone publishing them—is a risky enterprise, because those making them are left to reflect at leisure on their folly if they are shown subsequently to be wrong. Alternatively, a degree of smugness might be permitted the forecaster who gets it right.

The 65–74 age group is considered here for two main reasons. First, it is one of the World Health Organization (WHO) “pathfinder” groups [16], the others being 12-13, 20–24 and 35–44 years. These “pathfinder” groups are seen as useful indicators for describing dental disease in populations and for making interpopulation comparisons, because they represent important developmental epochs (life stages) in the natural history of oral conditions. Second, in common with other industrialised countries, New Zealand is undergoing a demographic transition: currently 13% of the population, the 65+ age group is predicted to comprise 23% of the population by 2051, while the proportion in the 0–14 age group will fall from its current 21% to 17% by then [17]. Occurring alongside that is what has been termed a “dental transition,” manifested in the falling prevalence of edentulism among older people as the edentulous “oldest old” die out and the incoming “youngest old” retire with at least some of their own teeth remaining. It is worth noting that the first of the baby boomer generation turned 65 during 2011, making that year somewhat of a demographic watershed for health planners and social policy-makers.

The national oral health surveys conducted in New Zealand in 1976 [12] and 1988 [13] reported the prevalence of edentulism in 65–74-year-olds to be 72.3% and 58.6%, respectively. These provided two time points from which projections were made subsequently [15]. In making those projections, the most likely shape of the decline had to be considered. It was unlikely to be a straight line, because that would have required edentulism prevalence among 65–74-year-olds to have been 100% once, and for it to reach 0% sometime in the decade beginning in 2040. Neither of those scenarios is likely to hold: a 100% edentulism rate would require everyone in that age group to have presented to a dentist for full clearance, and a 0% rate would need no one to have done so. Given the high rates of caries among New Zealand adults and the existing profound and largely unresponsive social and ethnic inequalities in the condition [14], the latter is highly unlikely. There will always be people whose oral problems are such that the most humane and clinically appropriate option is the complete removal of the natural dentition, followed by its prosthetic replacement. Thus, an alternative to a straight line needs to be considered, and the most likely candidate is a logistic decline (an S-shaped curve); this accommodates the two scenarios of the rate perhaps having once been near 100% and it approaching 0% sometime in the future, but never actually reaching either of those. Accordingly, the projection made for the year 2011—based upon the 1976 and 1988 estimates—for the 65–74 age group was 30.5%. At that time, there was little prospect of a third national oral health survey: the Government of the time had little interest in the public health approach, and a State-funded monitoring survey was but a pipe dream because the “hands-off,” free-market ethos which pervaded the public sector extended also to the Ministry of Health. Thus, making projections based upon the available data from the two previous surveys was the only option at that time.

A decade later, the political climate had changed, with three consecutive terms of a centre-left administration having (for the time being, at least) reset the philosophical parameters for the State's involvement in public health and its monitoring. Planning for the third national oral health survey got underway with the announcement of the available funding in 2006, and the data collection was implemented and completed during 2009. Publication of the technical report from that survey in late 2010 [14] allowed scrutiny of the validity of the projections made from the 1976 and 1988 data (Figure 1). As can be seen, the line of projection lies within the 95% confidence interval for the 2009 estimate (which was 29.6%). This suggests (a) that it was feasible to make medium-term projections from two relatively close time points and (b) that the decline in edentulism was indeed a logistic one. There appears to have been no other examination of this issue, and so the findings reported here are unique and should be helpful to those considering making projections from limited time-series data.

Although the data are not reported here, the 2009 survey observed ethnic and social differences in edentulism, with higher rates among Māori and those living in more deprived neighbourhoods [14]. This has clear public health implications, in that there is likely to be a continued widening of those social and ethnic differences, with predictable consequences for nutrition and quality of life among particular groups in New Zealand society.

What do these findings mean for dental public health and the provision of dental care? That edentulism continues to decline among older adults in New Zealand is encouraging, but any associated optimism should be tempered by awareness that caries-associated incremental tooth loss is highly prevalent [14] and having detrimental effects on people's lives [18]. The greater retention of teeth means more tooth surfaces at risk of caries and more periodontal sites at risk of inflammation. It is noteworthy that the prevalence of edentulism among 65–74-year-old Australians in 2004–06 (the period during which their second and most recent national oral health survey was conducted) was 20.3% [19], just over two-thirds of the New Zealand estimate for the same age group. While edentulism is falling in both countries, it is falling more quickly in Australia, where all of the State capitals are now fluoridated and there is greater Government involvement in the provision of routine dental care for low-income adults than in New Zealand. It is also possible that changes in the social influences on tooth retention have been more rapid in that country, but determining the scale and nature of any such changes is beyond the scope of this particular paper.

Sussex [1] pondered the implications for complete-denture prosthodontics and wondered whether the fall in edentulism prevalence would be offset by the increase in the absolute number of older people, so that the number requiring complete dentures might actually remain fairly constant. Computation of those numbers in the 65–74 age group in New Zealand (and assuming the validity of the 14% edentulism rate predicted for that age group by 2031 [15]) reveals that the absolute number of edentulous 65–74-year-olds will fall from 314,365 in 2011 to 76,300 in 2031. The latter is about one-quarter of the 2011 estimate and represents a substantial decrease in the absolute number of edentulous people in that age group. Thus, complete denture provision will become less common, and it is likely to be correspondingly more difficult due to dentists' lack of experience with it. Complete denture provision may eventually be left to prosthetists (also known as denturists or clinical dental technicians) in countries where they are permitted to practise. This is likely given the social and ethnic differences in edentulism which were observed in the 2009 survey [14].

Having fewer edentulous people will mean more complex prosthodontic challenges because of the sequelae of incremental (unplanned) tooth loss, such as drifting or over-eruption of the remaining teeth. There will be a need for more specialist prosthodontists and for general dentists to upskill in prosthodontics, as well as the associated areas of endodontics and periodontics.

In summary, edentulism continues to fall in New Zealand, a country which has had historically high prevalence rates. That fall has followed a logistic decline, with an earlier projection made from data two decades old turning out to be accurate. The findings also underline the usefulness of nationally representative oral health survey data.

## Figures and Tables

**Figure 1:**
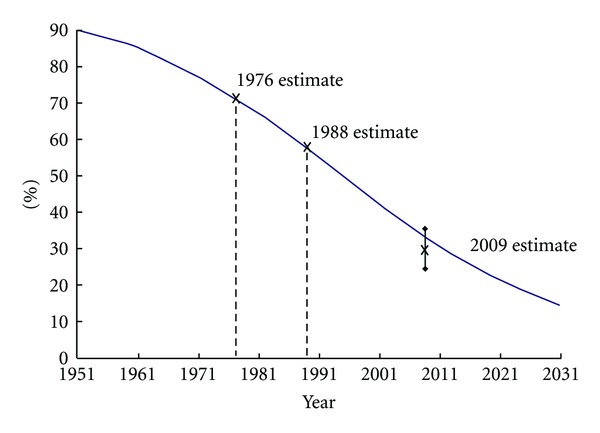
Decline in edentulism prevalence among New Zealand 65–74-year-olds, showing actual estimates from the 1976, 1988, and 2009 national oral health surveys, along with logistic line fitted in 1997 using the 1976 and 1988 estimates (2009 data point depicts 95% confidence interval).
